# Myo-inositol is a promising treatment for the prevention of ovarian hyperstimulation syndrome (OHSS): an animal study

**DOI:** 10.1007/s00404-015-3747-5

**Published:** 2015-05-20

**Authors:** Guluzar Arzu Turan, Fatma Eskicioglu, Oya Nermin Sivrikoz, Hakan Cengiz, Saban Adakan, Esra Bahar Gur, Sumeyra Tatar, Nur Sahin, Osman Yilmaz

**Affiliations:** Department of Obstetrics and Gynecology, Medical School, Sifa University, Sanayi cad no:7, 35100 Bornova, Izmir, Turkey; Department of Obstetrics and Gynecology, Medical School, Celal Bayar University, 45030 Manisa, Turkey; Department of Pathology, Medical School, Sifa University, Sanayi cad no:7, 35100 Bornova, Izmir, Turkey; Biostatistics and Medical Informatics, Medical School, Sifa University, Ankara Cad. No:45, 35100 Bayrakli, Izmir, Turkey; Molecular Medicine, Institute of Health Science, Dokuz Eylül University, Mithatpasa cad. No:1606 inciraltı yerleskesi, 35340 Balcova, Izmir, Turkey; Department of Laboratory Animal Science, Medical School, Dokuz Eylul University, Mithatpasa cad. No:1606 inciraltı yerleskesi, 35340 Balcova, Izmir, Turkey

**Keywords:** Ovarian hyperstimulation syndrome, Pigment epithelium-derived factor, Vascular endothelial growth factor, Metformin, Myo-inositol

## Abstract

**Purpose:**

To evaluate the efficacy of myo-inositol (MI) pretreatment in OHSS.

**Methods:**

In this experimental OHSS rat model, 42 immature Wistar albino female rats were divided into 6 groups: (1) the control group, (2) the ovarian stimulation group, (3) the OHSS group, (4) the OHSS + Metformin group, (5) OHSS + MI group, (6) OHSS + Metformin + MI group. OHSS was established after treatment with metformin and myo-inositol for 14 days, in the meanwhile the treatment of metformin and myo-inositol was also continued. All animals were killed 48 h after hCG administration and were compared in terms of vascular permeability, ovarian weight and diameter, ovarian VEGF, COX-2 and PEDF expression (immunohistochemistry), serum PEDF and estradiol (E2) levels.

**Results:**

Vascular permeability, VEGF and COX-2 expressions were reduced in animals treated with MI and/or metformin. While PEDF expression was increased in the groups taking metformin, there was no difference in PEDF expression in the group taking MI and OHSS group. There was no significant difference in serum PEDF levels between groups. Blood E2 levels were decreased in groups treated with MI or metformin compared to the OHSS group.

**Conclusions:**

Our data demonstrate that myo-inositol is effective in preventing OHSS, similar to metformin. Although the two drugs are thought to act through distinct mechanisms, there is no apparent benefit to co-treatment with both drugs in an animal model of OHSS. Administration of myo-inositol prior to IVF treatment may favor the control of ovulation induction. Further studies are necessary to elucidate the mechanism of action and further support our findings.

## Introduction

Ovarian hyperstimulation syndrome (OHSS) arises as an iatrogenic complication of assisted reproductive technology (ART). OHSS is defined by enlarged ovarian cysts and fluid leakage into the third space secondary to increased capillary permeability. Severe cases are potentially life-threatening and are characterized by acute respiratory distress syndrome (ARDS), hypovolemia, ascites, edema, and thrombosis [1].

Although the pathophysiology of OHSS is not fully understood, an increased vascular permeability due to the effect of hCG has been proposed. Vascular endothelial growth factor (VEGF) plays a key role in increased vascular permeability [2]. VEGF production is regulated by arachidonic acid metabolites and nitric oxide (NO) produced by nitric oxide synthase (NOS) and cyclooxygenase type 2 (COX-2) [3]. In relation to this, it has been shown that COX-2 inhibitors reduce ovarian expression of VEGF and COX-2 in the rat model of OHSS [4].

Moreover, pigment epithelium-derived factor (PEDF) is a potent angiogenic inhibitor in granulosa cells. Granulosa cells express and secrete PEDF and also, VEGF and PEDF have inverse effects in the metabolism [5].

OHSS prevention has been widely debated in the field of assisted reproductive techniques (ART). Identification of high-risk patients for OHSS before treatment and the initiation of effective preventative interventions are essential for safety in ART. Patients with polycystic ovarian syndrome are at an extreme risk for the development of OHSS [6].

Polycystic ovary syndrome (PCOS) is a metabolic and endocrine disorder affecting 5–10 % of reproductive-age women. PCOS is characterized by hyperandrogenism and chronic oligo- or anovulation. Insulin resistance, compensatory hyperinsulinemia and central obesity are associated with PCOS and play a key role in the pathogenesis of hyperandrogenism and anovulation [7].

Insulin-sensitizing agents are often recommended in PCOS patients to treat metabolic imbalances. In addition to treating the metabolic disorder, increasing the fertility and reducing the risk of OHSS are of importance [8–10].

Metformin (*N*,*N*-dimethylbiguanide) is an insulin-sensitizing agent that is widely used in the management of type II diabetes. Although mechanism of action of metformin is not clearly understood, metformin is known to enhance the effect of insulin through peripheral insulin receptors. In addition, metformin promotes fatty acid oxidation and glucose uptake while inhibiting glucose production. Metformin decreases androgen levels in patients with PCOS, improving the frequency of ovulation and menstrual cycles [8, 11]. Metformin also reduces OHSS risk in IVF patients; this is yet another contributing factor to its clinical significance [10].

Myo-inositol (MI) is another widely used insulin-sensitizing agent with increasing popularity in recent years. MI acts through the secondary messenger system to modulate metabolic enzymes in a manner that is similar to the effects of insulin, enhancing insulin sensitivity [12]. MI is effective in improving metabolic and hormonal balance in PCOS patients, similar to other insulin-sensitizing agents [13]. Supportive treatment with MI promotes spontaneous ovarian activity and has positive effects in the treatment of infertility [14].

Results of several studies support the possibility that MI may serve as a first-line treatment in PCOS patients, as it is simple, safe, and effective. Whether or not MI is effective in preventing OHSS is of importance when treating PCOS patients, especially when considering that they often present for reproductive therapy. However, no previous study has evaluated the relationship between MI and OHSS. In addition, the mechanism of action of metformin and MI are distinct but have not been fully elucidated. Therefore, it is important to formally evaluate the efficacy of inositol in preventing OHSS relative to metformin and to investigate the potential benefits of combinatorial treatments using both drugs.

The aim of this study is to investigate whether MI is effective in preventing OHSS in a rat model, and in addition, to investigate additive effects of MI treatment when used in combination with metformin.

## Materials and methods

### Animals

Immature female Wistar albino rats weighing 30–60 g were obtained from the animal laboratory of Dokuz Eylul University. The animal protocol was reviewed and approved by Dokuz Eylul University Local Ethics Committee on Animal Experiments in accordance with the NIH Guide for the Care and Use of Laboratory Animals, Institute of Laboratory Animal Resources (National Research Council, Washington, D.C.). All animals were maintained at 22 ± 2 °C, 55 % humidity, under 12/12 h day/night photoperiods and they were fed ad libitum. Animals were housed 3–4 per cage under standard laboratory conditions.

A total of 42 immature Wistar albino female rats (22 days old) were randomly divided using a random number table into 6 groups: (1) the control group (*n* = 7), which received no treatment; (2) the ovarian stimulation group (*n* = 7), which received 10 IU of pregnant mare serum gonadotropin (PMSG) (Folligon, 5 × 1000 IU + Diluent, MSD, Animal Health, Intervet International, Netherlands) on the 39th day and 10 IU of human chorionic gonadotropin (hCG) (Chorulon, 5x1500 IU + Diluent, MSD, Animal Health, Intervet International, Netherlands) 48 h later (day 41) to mimic routine ART protocols; (3) the OHSS group (*n* = 7), which received 50 IU of PMSG daily from days 37 to 40 and 30 IU hCG on day 41 to induce OHSS; (4) the OHSS + Metformin group (*n* = 7); (5) OHSS + MI group (*n* = 7); (6) OHSS + Metformin + MI group. Groups 4, 5 and 6 underwent the same hormonal stimulation protocol as the OHSS group in addition to treatment with 50 mg/kg/d metformin (Glucophage 500 mg tablet, Merck, Turkey) and/or 75 mg/kg/d MI (Inofolic 1 gr, ITF, Turkey) for 20 consecutive days from days 22 to 43 (Table 1). In the group receiving both metformin and MI, there was a 10 min interval between the administration of the two drugs. We applied 2 weeks of metformin and MI treatment before ovulation induction similar to OHSS rat model study of Elia et al [15].

**Table 1:**
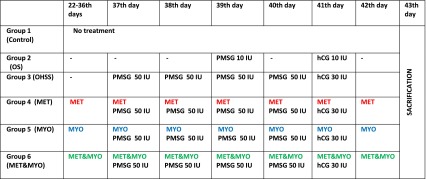
Timetable of treatment

All hormonal treatments were administered subcutaneously, while metformin and MI were administered orally. All groups were killed at 48 h after hCG administration on day 43. The groups were compared in terms of vascular permeability; ovarian weight; ovarian diameter; vascular endothelial growth factor (VEGF); pigment epithelium-derived factor (PEDF) and cyclooxygenase-2 (COX-2) expression (immunohistochemistry) in the ovarian tissue; differences in pigment epithelium-derived factor (PEDF) and estradiol (E2) levels in the serum using the Kruskal–Wallis and Mann–Whitney *U* tests.

Experiments were performed 48 h after the hCG injections. All experimental animals were weighed prior to killing and anesthetized with a subcutaneous injection of 5 mg/kg xylazine and 35 mg/kg ketamine (Alfamine 10 %, Alfasan International B.V., Netherlands) and (Alfazyne 2 %, Alfasan International B.V., Netherlands). The neck was dissected and the jugular vein was injected with 2 mL of 5 mM Evans Blue (Sigma-Aldrich, USA) to evaluate vascular permeability. After a 30 min waiting period, 5 mL 0.9 % isotonic saline solution was injected into the peritoneum and abdominal massage was performed for 30 s. The peritoneal fluid was subsequently aspirated from the abdominal cavity and collected in tubes containing 0.05 ml 0.1 N NaOH. The animals were euthanized after obtaining cardiac blood samples to be used for PEDF and hormone (estradiol) assays. Finally, the ovaries were excised.

### Ethical approval

All of the experimental procedures were approved by Dokuz Eylul University Local Ethics Committee on Animal Experiments (101/2013).

### Evaluation of vascular permeability

Collected peritoneal fluids were centrifuged at 900 g for 12 min. Evans Blue staining was quantified at 600 nm using a Shimadzu UV-1800 UV–Visible spectrophotometer. Vascular permeability was expressed as the concentration of Evans Blue (mM) per 100 g body weight.

### Immunohistochemistry and histopathological evaluation

The ovarian tissues were stained using immunohistochemical methods to evaluate VEGF, COX-2 and PEDF expression. The ovaries were fixed in 10 % buffered formalin. Ovarian tissues were embedded in paraffin blocks after a follow-up procedure. Paraffin blocks were cut into 4-µm sections and deparaffinized. One section per block was stained with hematoxylin and eosin (H&E), and the remaining sections were stained with VEGF antibody (Monoclonal Mouse Anti-Human VEGF, Clone VG1, Dako, Denmark), COX-2 antibody (Monoclonal Mouse Anti-Human COX-2, Clone CX-294, Dako, Denmark) and PEDF antibody (HPA005825 Anti-SERPINF1, Atlas Antibodies, Sweden) using an immunohistochemistry DakoCytomation Autostainer. The glial cell preparation was evaluated as a control for antibody staining of PEDF expression. The percentage of stained luteinizing granulosa cells and the intensity of staining were quantified by light microscopy (400X, Olympus BX53, Tokyo, Japan). Staining intensity was quantified as follows: 0 (no staining: no cells), 1 (minimal staining: 1–25 % of cells), 2 (mild staining: 26–50 % of cells) or 3 (intense staining: >50 % of cells). Ovarian diameter was measured in all sections.

### PEDF assay

Serum PEDF was quantified (pg/ml) using a commercially available enzyme-linked immunosorbent assay (ELISA) according to the manufacturer’s instructions (Cusabio Biotech, Rat pigment epithelium-derived factor ELISA kit, Cat. no: CSB-E08819r, Hubei Province, China).

### Hormonal assay

Measurement of plasma estradiol (E2) levels was performed at the Biochemistry Laboratory of Sifa University Hospital by means of chemiluminescence with the Roche Cobas analyzer. All samples were measured at the same time to minimize error. E2 serum results are expressed as picograms per milliliter of serum (pg/ml).

### Statistical analyses

Statistical analyses were performed in R using Rstudio version 0.98.501. Both analytical (Kolmogorov–Smirnov and Shapiro–Wilk tests) and visual methods (histograms and probability plots) were used to evaluate the distribution of continuous variables. Descriptive analyses are presented as mean and standard deviation for normally distributed variables (body weight before; body weight after; delta body weight; vascular permeability; ovarian weight; ovarian diameter; percentage and staining intensity of VEGF, COX-2 and PEDF; serum PEDF and E2 levels). The Mann–Whitney *U* test was applied when data did not follow a normal distribution. The Kruskal–Wallis H test was applied for the comparison of three or more groups under non-parametric conditions. A *p* value <0.05 was considered statistically significant.

## Results

### Weight gain

There was no significant difference in weight gain between the control group and the treatment group. The group treated with metformin exhibited the smallest gain in weight among all the experimental groups. Weight increase was significantly reduced in the metformin treatment group relative to the MI treatment group (*p* = 0.015).

### Vascular permeability

**Fig. 1:**
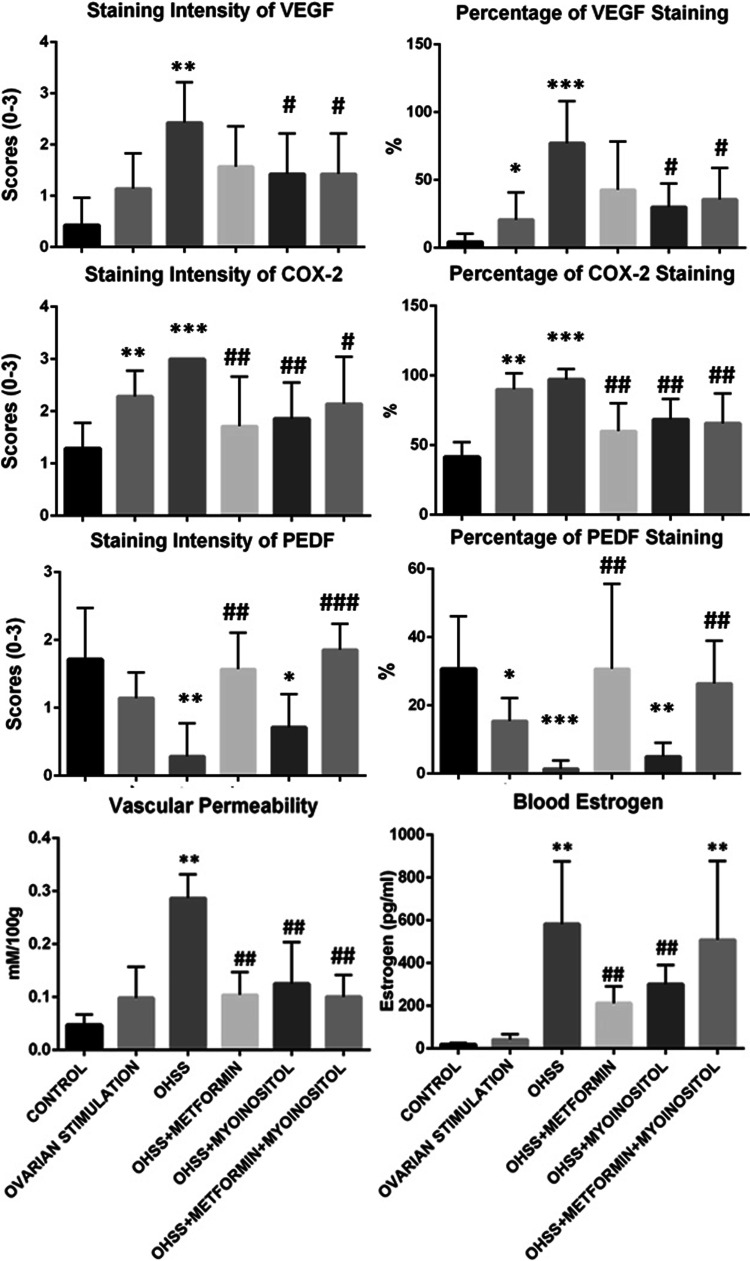
*p < 0.05, **p < 0.01, ***p = 0.001 respect to control group; ^**#**^ *p* < 0.05, ^**##**^ *p* < 0.01, ^**###**^ *p* = 0.001 respect to OHSS group

There was no significant increase in vascular permeability in the ovarian stimulation group (2nd group) compared to the control group. However, there was a significant increase in all OHSS groups (group 3, 4, 5 and 6). Vascular permeability was significantly decreased in group 4, 5 and 6 receiving metformin and/or MI compared to the OHSS group (*p* = 0.002, *p* = 0.002, *p* = 0.002, respectively). Vascular permeability was similar in the ovarian stimulation group (group 2) compared to groups 4, 5 and 6 (*p* = 0.85, p = 0.57, *p* = 0.95, respectively). There was no difference in vascular permeability among groups 4–6 (Table 2; Fig. 1).

**Table 2:** Characteristics of the study groups

Characteristics	Group 1 control (*n* = 7)	Group 2 ovarian stimulation (*n* = 7)	Group 3 OHSS (*n* = 7)	Group 4 metformin (*n* = 7)	Group 5 Myo-inositol (*n* = 7)	Group 6 Metformin & Myo-inositol (*n* = 7)
Body weight before (g)	43.1 ± 10.3	47.4 ± 11.2	34.1 ± 3.5*	33.2 ± 1.0	31.5 ± 1.5	33.9 ± 1.4
Body weight after (g)	135.1 ± 15.4	132.0 ± 14.2	113.9 ± 4.4**	99.7 ± 21.1	118.4 ± 6.5^**##**^	112.6 ± 6.5
Delta body weight (after–before)	92.0 ± 7.2	84.6 ± 6.8	79.8 ± 5.3*	66.5 ± 21.0	86.9 ± 6.2^**##**^	78.9 ± 6.7
Vascular permeability (Evans Blue mM/100 g)	0.05 ± 0.02	0.10 ± 0.06	0.29 ± 0.05**	0.10 ± 0.05^**##**^	0.13 ± 0.10^**##**^	0.10 ± 0.04^**##**^
Ovarian weight (µg)	49.4 ± 85	153.3 ± 24**	206 ± 37**	132 ± 63^**#**^	190 ± 57	179.6 ± 38
Ovarian diameter (mm)	3.6 ± 0.8	6.4 ± 0.5***	8.0 ± 0.8***	6.0 ± 2.2	5.4 ± 1.1^**##**^	6.6 ± 1.7
VEGF staining percentage (%)	4.3 ± 6.1	20.7 ± 20.1*	77.1 ± 30.9***	42.9 ± 35.5	30.0 ± 17.3^**#**^	35.7 ± 23.2^**#**^
VEGF staining intensity (0–3)	0.4 ± 0.5	1.1 ± 0.7	2.4 ± 0.8**	1.6 ± 0.8	1.4 ± 0.8^**#**^	1.4 ± 0.8^**#**^
COX-2 staining percentage (%)	41.4 ± 10.7	90.0 ± 11.6**	97.1 ± 7.6***	60.0 ± 20.0^**##**^	68.6 ± 14.6^**##**^	70.5 ± 23.6^**##**^
COX-2 staining intensity (0–3)	1.3 ± 0.5	2.3 ± 0.5**	3.0 ± 0.0***	1.7 ± 1.0^**##**^	1.9 ± 0.7^**##**^	2.1 ± 0.9^**#**^
PEDF staining percentage (%)	30.7 ± 15.4	15.4 ± 6.7*	1.4 ± 2.4***	30.7 ± 24.9^**##**^	5.0 ± 4.1	26.4 ± 12.5^**##**^
PEDF staining intensity (0–3)	1.7 ± 0.8	1.1 ± 0.4	0.3 ± 0.5**	1.6 ± 0.5^**##**^	0.7 ± 0.5	1.9 ± 0.4^**###**^
PEDF (pg/ml)	15.6 ± 3.1	16.5 ± 4.1	14.6 ± 2.9	10.76 ± 2.7^**#**^	12.1 ± 1.4	12.7 ± 3.8
Estrogen (pg/ml)	18.4 ± 8.0	42.4 ± 25.3	583.3 ± 292.0**	213.3 ± 77.3^**##**^	300.8 ± 90.7^##^	508.9 ± 367.8

Values are expressed as mean ± SD and *p* values are determined by Mann–Withney *U*, followed by Kruskal–Wallis test. * *p* < 0.05, ** *p* < 0.01, *** *p* = 0,001 respect to control group; # *p* < 0.05, ## *p* < 0.01, ### *p* = 0.001 respect to OHSS group

### Ovarian weight

Ovarian weight increased in all groups relative to the control group. Ovarian weight was significantly reduced in the metformin treatment group compared to the OHSS group (*p* = 0.013).

### Ovarian diameter

Ovarian diameter was increased in all treatment groups relative to the control groups. The increase in ovarian diameter was smallest in the MI treatment group (group 5).

### Immunohistochemistry and histopathological evaluation

**Fig. 2:**
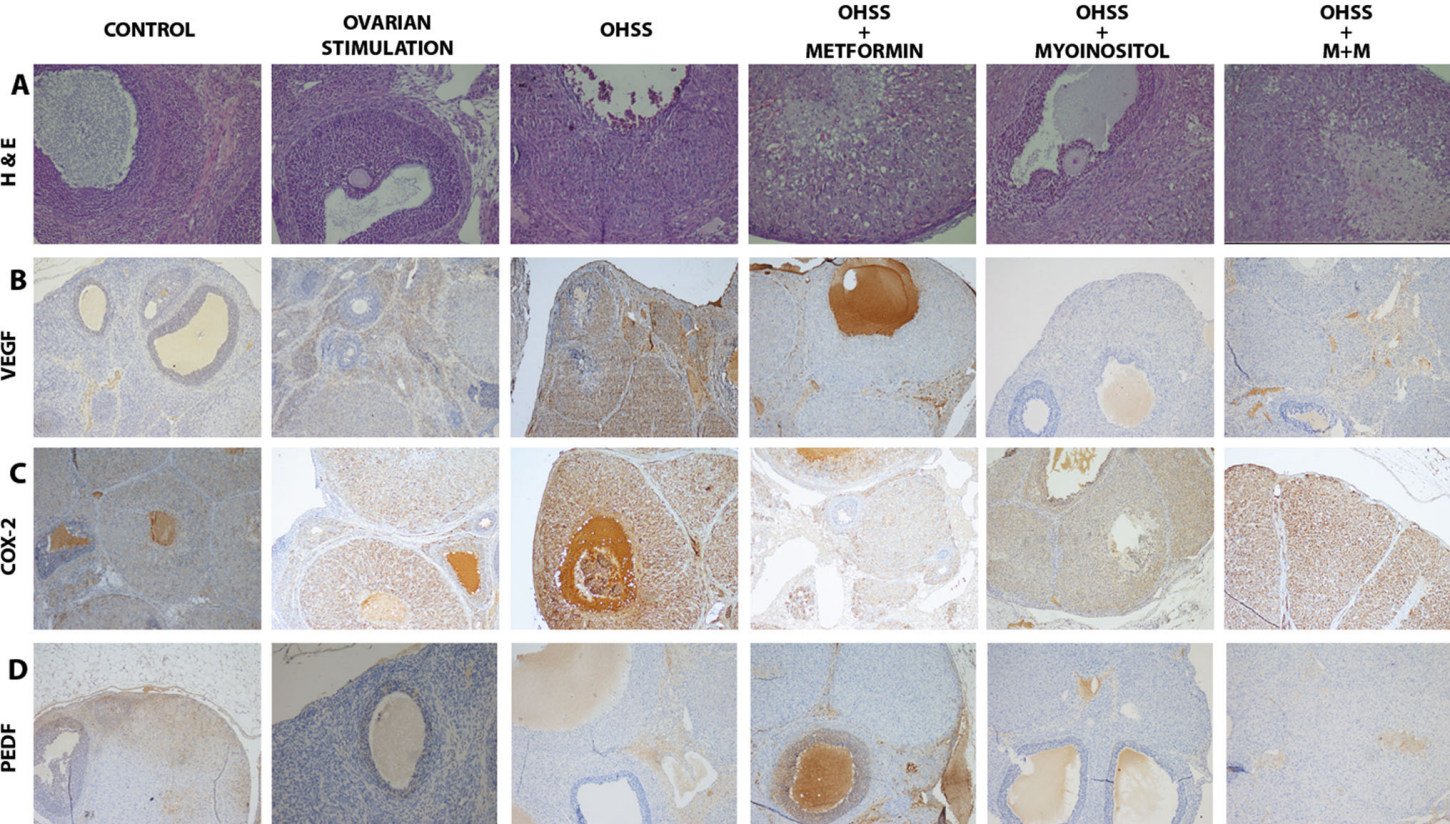
**a** Intensive luteinization of the granulosa cells in the metformin treatment group and the metformin and myo-inositol co-treatment group (hematoxylin & eosin staining). Luteinization is reduced in granulosa cells from the myo-inositol treated animals relative to metformin-treated animals (magnification ×200). **b** VEGF staining immunohistochemically was increased in all groups receiving ovarian stimulation treatment. There was a significant decrease in VEGF in groups 5 and 6 relative to the OHSS group (magnification ×100). **c** COX-2 staining was also increased in all groups receiving ovarian stimulation. There was a decrease in COX-2 staining in groups 4–6 relative to the OHSS group (magnification ×100). (D) PEDF staining was highest in the control group. There was a significant decrease in the OHSS group. There was a significant increase in PEDF in both groups receiving metformin (groups 4 and 6). Similar PEDF stainings in the MI and OHSS groups were remarkable (magnification ×100)

An increase in luteinization of granulosa cells was observed in the OHSS groups relative to the control group in the hematoxylin and eosin-stained sections (Fig. 2). Similarly, luteinized granulosa cells were markedly increased in the metformin group and metformin + MI group. However, luteinization was noticeably less in the group receiving MI alone compared to other OHSS groups (groups 3, 4, and 6).

### VEGF

Staining intensity and percentage of VEGF were increased in all groups receiving ovarian stimulation treatment (Table 2; Figs. 1, 2). There was no significant difference among groups 4–6. There was a significant decrease in VEGF in groups 5 and 6 relative to the OHSS group. VEGF was decreased in the metformin group; however, the difference was not significant (percentage *p* = 0.076, intensity *p* = 0.067).

### COX-2

COX- 2 staining percentage was increased in all groups receiving ovarian stimulation (group 3, 4, 5 and 6). There was no difference in COX-2 staining among groups 4–6. There was a significant decrease in COX-2 staining in groups 4–6 relative to the OHSS group (Table 2; Figs. 1, 2).

### PEDF

PEDF staining intensity and percentage positive cells were highest in the control group. There was a significant decrease in the OHSS group (Intensity *p* = 0.004, percentage *p* = 0.001). There was a significant increase in PEDF in both groups receiving metformin (groups 4 and 6) (Table 2; Figs. 1, 2). On the other hand, it was remarkable that PEDF staining in the group receiving MI alone was similar to the OHSS group (intensity *p* = 0.122, percentage *p* = 0.08).

### PEDF assay

When differences in serum PEDF concentrations were assessed using the Kruskal–Wallis test, there was a significant difference among the experimental groups (*p* = 0.022). However, when groups were compared to each in a pairwise manner using the Mann–Whitney *U* test, PEDF was significantly reduced in group 4 compared to first three groups and in group 5 relative to groups 1 and 2.

### Estrogen assay

E2 concentration was increased significantly in all groups undergoing OHSS treatment relative to the control group. E2 concentration was significantly decreased in the groups receiving metformin alone or MI alone compared to the OHSS group (metformin *p* = 0.004, MI *p* = 0.009). E2 concentration was similar to the OHSS group in the group receiving both metformin and MI (*p* = 0.406). There was no difference in E2 concentration between the metformin (4) and MI (5) groups (Table 1; Fig. 1).

## Discussion

The results of the present study demonstrated that MI and metformin are effective in reducing the severity of OHSS when used alone or in combination. There was no additional benefit to treat with the two drugs in combination. Metformin and MI suppress vascular permeability and VEGF expression which is the primary driver of OHSS pathogenesis.

PEDF down-regulates VEGF expression and has anti-vasopermeability, anti-thrombogenic and anti-angiogenic properties [16]. Chuderland et al. [17] have demonstrated that replacement of PEDF could be effective in treatment in mice with OHSS. In our immunohistochemical examination of ovarian tissues, we found the highest levels of PEDF expression in the control group and the lowest PEDF expression in the OHSS group. There was an increase in PEDF in groups receiving metformin (groups 4 and 6); in both groups OHSS severity (VEGF expression and vascular permeability) was reduced. Surprisingly, there was no PEDF increase in the group receiving MI, and the PEDF was similar to the OHSS group.

COX-2 expression in the present study was consistent with the findings of Elia et al. [15] investigating the effects of metformin in the rat model of OHSS. COX-2 expression increased with increasing OHSS severity. There was a significant decrease in COX-2 expression in all groups receiving MI and/or metformin. The fact that there was no increase in PEDF in the group receiving MI alone unlike metformin receiving groups suggests that MI alters COX-2 expression but not through PEDF.

Although there was a statistically significant difference among the experimental groups in serum PEDF concentrations as measured by ELISA, these differences were not clinically meaningful. Therefore, changes in the PEDF expression in the tissue may be reflected in the systemic circulation subsequently.

In the histopathological examination of H&E stained preparations, luteinisation of the granulosa cells was reduced in the group receiving only MI (group 5) unlike the metformin groups (group 4 and 6) when compared to the OHSS group. The results of the metformin and MI co-treatment arm (group 6) of the experiment were particularly interesting. Interactions between the two drugs may cause these results and the mechanism of action needs to be clarified. Two clinical studies involving metformin and MI co-treatment have been previously published [18, 19]. Both of these studies suggest potential benefits associated with MI co-treatment. Neither of these studies mentioned any similar results found in our study.

Both metformin and MI, when used alone, reduce the E2 concentration. Although there was no statistically significant difference, the E2 concentration in the co-treatment group (group 6) was higher than the metformin (group 4) and MI (group 5) treatment groups; and intense luteinisation of the granulosa cells was observed in both the metformin treatment group (group 4) and the metformin and MI co-treatment group (group 6). These results suggest that beneficial effects of MI may be masked by the metformin in co-treatment group.

The effects of metformin on OHSS have been studied for at least 10 years. Numerous clinical and experimental studies have indicated the positive effects of metformin on reproductive health. Metformin has been demonstrated to inhibit the production of androgens in the ovaries and reduce hyperinsulinemia. Metformin is often preferred especially for correcting insulin insensitivity, promoting weight loss while reducing the risk of gestational diabetes and inducing spontaneous ovulation [20]. Moreover in ART, metformin is known to enhance oocyte quality along with the preventive effects on OHSS which is often seen among PCOS patients. A Cochrane systematic review published in 2014 reported that metformin increased clinical pregnancy rates and reduced the OHSS risk, with no effect on live birth rate [21].

MI is a pharmacological agent whose effects have been recently discovered in comparison to metformin. The majority of studies proposing positive effects of MI in PCOS patients have been published within the last 5 years. MI increases insulin sensitivity, induces spontaneous ovulation, suppresses LH production, and increases oocyte quality during IVF treatment like metformin [22, 23]. MI also regulates FHS signals in PCOS patients [13]. Papaleo et al. [24] reported that MI increased oocyte quality in ICSI cycles and observed that E2 levels on hCG day in the individuals receiving MI were lower. Based on this finding, the authors commented that MI might reduce the risk of OHSS. However, no study has addressed this possibility as of yet.

We investigated the effects of MI on OHSS for the first time in the present study. We demonstrated that inositol was effective in preventing OHSS, similar to metformin. In addition, we investigated the possible benefits of the combination of metformin and MI. We concluded that there was no additional advantage to use in combination and the effects of the two drugs may overlap substantially.

Cabergoline is an effective and widely used agent in patients with high OHSS risk. Unlike insulin-sensitizing agents, the effects of cabergoline are through secondary prevention [25]. We did not evaluate cabergoline in the present study since mechanism of action of cabergoline is completely distinct [26].

Although the results of the present study suggest that MI has substantial clinical utility, clinical studies that support these findings are needed to recommend use of MI for OHSS prevention. The effects of MI on live birth rate, early pregnancy loss rate, pregnancy after improvement in OHSS should be evaluated relative to the current clinical standard, metformin. Also the finding that luteinisation was reduced in granulosa cells of animals receiving MI relative to those receiving metformin results in the necessity of evaluation of luteal phase.

MI is comparable to metformin in preventing OHSS; however MI is significantly more expensive than metformin. Since there is no evidence that MI is superior to metformin in the treatment of OHSS, patients needs, medication side effects, medication costs, and other factors may influence the physicians decision to prescribe one drug over another. Monitoring and appropriate treatment are important in patients undergoing controlled ovarian hyperstimulation. Antagonist protocol with agonist trigger and total freezing remains the most effective method for preventing OHSS in good clinical practice [27, 28].

### Limitations of our study

We administered metformin and MI together in group 6 with the assumption that the two drugs would act through distinct mechanisms. However, we had to administer the two drugs within a 10 min period because of physical limitations. The interaction of the two drugs could be the reason why we did not see our expected result. However, we have no data regarding the potential pharmaceutical interaction of metformin and MI.

The measurement of serum E2 levels validated the OHSS model and allowed for the evaluation of therapeutic interventions. However, the reduction in granulosa cells luteinisation in the MI group highlights the limitations regarding the lack of data on progesterone expression. We found that MI is effective in prevention of OHSS; however, more research is needed to elucidate its role in the luteal phase.

## Conclusion

MI is effective in preventing OHSS, and is comparable to metformin in clinical efficacy. Although these two drugs are thought to act through distinct mechanisms, there is no benefit associated with co-treatment in our model system. The effects of MI may be masked by metformin co-treatment.

Administration of MI prior to IVF treatment may help to improve the internal balance of the ovary and to promote a more controlled ovarian response. There remain several issues where clarification is necessary regarding the mechanism of OHSS. Further investigations to clarify the pathophysiology of OHSS and clinical settings to support these findings are needed.
